# Endpoints in Vital Signs as a Useful Tool for Measuring Successful Needle Decompression After Traumatic Tension Pneumothorax: An Analysis of the National Emergency Medicine Information System Database

**DOI:** 10.7759/cureus.30715

**Published:** 2022-10-26

**Authors:** Raymond I Okeke, Thomas Hoag, John T Culhane

**Affiliations:** 1 General Surgery, Saint Louis University School of Medicine, Saint Louis, USA; 2 Trauma Surgery, Saint Louis University School of Medicine, Saint Louis, USA; 3 Surgery, Saint Louis University School of Medicine, Saint Louis, USA

**Keywords:** database, vital signs, tension pneumothorax on pocus, tension pneumothorax, major trauma

## Abstract

Background

Needle decompression is a useful tool in the pre-hospital setting for treating tension pneumothorax. However the specific improvements in vital signs that determine a successful decompression are only reported in a few studies and Emergency Medical Services (EMS) self-reported assessments of improvement are more commonplace. We hypothesize that EMS reports may exaggerate improvement when compared to objective vital sign changes.

Methodology

This is a retrospective cohort study using the National Emergency Medicine Information System (NEMSIS) for the year 2020. Vital signs recorded as objective endpoints include systolic blood pressure (SBP), pulse (HR), respiratory rate (RR), and oxygen saturation (SpO_2_). Univariate analysis was performed using the t-test for continuous variables and the chi-square test for categorical variables.

Results

A total of 8,219 calls were included in the sample size analyzed. Most patients were white (2,911, 35.4%) and male (6,694, 81.4%). Abnormal vitals recorded as indications for needle decompression included SBP <100 mmHg, HR <60 or >100 beats/minute, RR <12 or >20 breaths/minute, and SpO_2_ <93%. Statistically significant improvements were seen in the number of abnormal vital signs after the procedure. The percentage of improvement was higher in the EMS self-reported assessment than in objective findings for oxygen saturation and SBP.

Conclusions

Our analysis shows objective improvement of hypoxia and hypotension after field needle decompression, supporting the efficacy of the procedure. The improvement based on vital sign change is modest and is less than that reported by EMS assessment of global improvement. This represents a target for quality improvement in EMS practice.

## Introduction

Traumatic tension pneumothorax is the second most common injury from chest trauma in the United States [1,2]. Thoracic trauma can be categorized into blunt or penetrating, both of which can cause simple pneumothorax. When the condition progresses to involve hemodynamic changes, it can become a tension pneumothorax [2]. Tension pneumothorax is a life-threatening condition that requires pre-hospital intervention by Emergency Medical Services (EMS) personnel to prevent hypoxia and circulatory arrest [3]. The primary goal is to minimize the time between injury and medical care [4]. Needle decompression can alleviate tension pneumothorax in the pre-hospital setting [1]. EMS personnel may face field conditions that make clinical signs of tension pneumothorax difficult to assess, and even with accurate assessment, precise indications for needle decompression are not always clear.

The American College of Surgeons Advanced Trauma Life Support (ATLS) and Prehospital Trauma Life Support (PHTLS) recommend needle decompression as the initial treatment for tension pneumothorax [5,6]. However, neither gives specific quantitative guidelines for performing these procedures or the endpoints to indicate success [1,2]. Additionally, needle decompression is an invasive procedure with the potential for severe injury to both the chest wall and thoracic viscera, and for most paramedics, it is a rare procedure with a steep learning curve. Thus, the variability and inconsistency between EMS protocols present a problem for the appropriate diagnosis and treatment of tension pneumothorax in the pre-hospital setting. Quality improvement in the form of continued training and ongoing assessment of risk and benefit is key to safe practice and requires an accurate measure of outcomes. Current measurements such as EMS self-assessment are limited by unclear criteria and a lack of long-term outcome data.

This study aims to objectively evaluate the success of pre-hospital needle decompression in the treatment of tension pneumothorax using improvement in average vitals and compare this improvement to that reported by EMS personnel assessment. We hypothesize that the EMS personnel-reported global improvement may be currently exaggerated when compared to objective vital sign changes.

## Materials and methods

This is a retrospective cohort study using the National Emergency Medicine Information System (NEMSIS) for the year 2020. All emergency transport calls were reviewed for field pleural decompression. All patients who underwent field decompression for tension pneumothorax were included. Baseline characteristics for the population were described. Systolic blood pressure (SBP), heart rate (HR), respiratory rate (RR), and oxygen saturation (SpO_2_) were analyzed before and after the procedure. Abnormal vitals recorded as indications for needle decompression included SBP <100 mmHg, HR <60 or >100 beats/minute, RR <12 or >20 breaths/minute, and SpO_2_ <93%. If at least one measurement of the parameter was not available before and after the procedure, the patient was omitted from that part of the analysis. Paired t-test was used to test for the significance of the difference of means of continuous variables before and after the procedure. A binomial test was used to test for the significance of the number of patients with vitals abnormal before and after the decompression procedure. The chi-square test was used to test for the significance of the number of patients improved based on objective vital signs versus EMS assessment.

All statistical analysis was conducted with SPSS statistics, version 27.0 (IBM Corp., Armonk, NY, USA). The SPSS Python plug-in and standalone Python were used for selected data processing tasks. Each procedure is linked to an EMS call with a call identification key which allows us to identify every call involving a pleural decompression. We constructed a dictionary of these keys which we used to retrieve all vital signs associated with each EMS call involving pleural decompression. Variables from the various other NEMSIS data files were extracted and added to a Python dictionary to report the baseline characteristics and secondary outcomes.

NEMSIS defines the success of the procedure as a successful pleural entry. Success and patient response are coded per attempt. We convert this to overall success per EMS call. Separate procedural attempts during the same call may have different success and response results. If any attempt is successful, we code the procedure as successful for the call. If any attempt leaves the patient worse, we code the procedure at the call level as worse. If the response to any attempt is improved and there is no recorded response of worsened condition, the overall call procedure is classified as improved.

To determine whether abnormal pre-procedure vital signs improved or worsened we calculated the average vital sign parameters before and after pleural decompression. We counted the patients with abnormal vital signs before and after the procedure, classifying the response as normalized, unchanged, or becoming abnormal. The number of patients with abnormal vital signs before and after the procedure was compared as a measure of the overall outcome.

We analyzed changes in vitals that did not cross the threshold between normal and abnormal but might still represent an improvement or worsening. If the average value before the procedure is abnormal but becomes closer to normal than before the procedure, it is classified as improved. Conversely, if the average value before the procedure is abnormal but deviates even farther from the reference range after the procedure, it is classified as worsened.

## Results

A total of 8,219 patients were included in the sample size analyzed. In total, 2,911 (35.4%) patients were White, the most recorded ethnicity (Table 1). A total of 6,694 (81.4%) patients were male (Table 2). In total, 6,861 (83.5%) patients had a trauma-related injury prior to EMS arrival (Table 3), and 2,141 (26%) patients had a cardiac arrest prior to EMS arrival (Table 4). A total of 11,669 complete needle decompression procedures were performed during the study period (Table 5). In total, 10,881 (93.25%) procedures involved one attempt (Table 5). A total of 8,571 (73.5%) procedures were performed by paramedics (Table 6). Vital signs recorded as objective endpoints for the success of needle decompression included SBP, HR, RR, and SpO_2_. Abnormal vitals recorded as indications for needle decompression included SBP <100 mmHg, HR <60 or >100 beats/minute, RR <12 or >20 breaths/minute, and SpO_2_ <93%. Mean changes to vital signs were recorded before and after the procedure with p-values (Tables 7, 8). SpO_2_ showed the highest improvement with a drop from 2,070 (67.2%) abnormal patients before the procedure to 1,289 (41.8%) after the procedure (Table 8). EMS assessed 10,708 (91.8%) procedures as successful (Table 9) and 4,815 (41.2%) patients as improved (Table 10). SpO2 was measured as improved in 1,627 (52.8%) patients compared to 1,181 (56.6%) patients, as reported by EMS personnel (Tables 11, 12).

**Table 1:** Frequency and percentage of needle decompression procedures by race.

Race	Frequency	Percentage (%)
White	2,911	35.4
Black	1,127	13.7
Hispanic	446	5.4
Other	112	1.4
Not recorded	3,623	44.1
Total	8,219	100.0

**Table 2:** Frequency and percentage of needle decompression procedures by gender.

Gender	Frequency	Percentage (%)
Female	1,459	17.8
Male	6,694	81.4
Not applicable	6	0.1
Not recorded	48	0.6
Unknown (unable to determine)	12	0.1
Total	8,219	100.0

**Table 3:** Frequency and percentage of trauma-related injury in calls ending in needle decompression procedures. EMS: Emergency Medical Services

Traumatic mechanism	Frequency	Percentage (%)
Yes	6,861	83.5
No	882	10.7
Not recorded	476	5.8
Total	8,219	100.0

**Table 4:** Frequency and percentage of cardiac arrests in calls ending in needle decompression procedures. EMS: Emergency Medical Services

Cardiac arrest	Frequency	Percentage (%)
Yes, prior to EMS arrival	2,141	26.0
Yes, after EMS arrival	1,124	13.7
No	3,601	43.8
Not recorded	1,353	16.5
Total	8,219	100.0

**Table 5:** Number of attempts per needle decompression procedure.

Number of attempts	Number of procedures	Percentage (%)
1	10,881	93.25
2	200	1.71
3	14	0.12
4	3	0.03
5	5	0.04
10	3	0.03
Not recorded	563	4.82
Total	11,669	100

**Table 6:** Frequency and percentage of providers performing the needle decompression procedure. EMS: Emergency Medical Services; RN: registered nurse; NP: nurse practitioner

Role of provider	Frequency	Percentage (%)
Paramedic	8,571	73.5
Other EMS	586	5.0
RN/NP	590	5.0
Physician	54	0.5
Other	1,868	16.0
Total	11,669	100.0

**Table 7:** Mean vital signs before and after the needle decompression procedure. SBP: systolic blood pressure (mmHg); HR: heart rate (beats/minute); RR: respiratory rate (breaths/minute); SpO_2_: oxygen saturation (%)

Vital sign	Number of calls	Mean before procedure	Mean after procedure	P-value
					SBP	2,746	116.12	119.21	<0.001
HR	3,900	99.97	98.14	<0.001
RR	4,028	20.31	18.68	<0.001
SpO_2_	3,082	85.88	90.35	<0.001

**Table 8:** Changes in the measured vital signs before and after the procedure. SBP: systolic blood pressure (mmHg); HR: heart rate (beats/minute); RR: respiratory rate (breaths/minute); SpO_2_: oxygen saturation (%)

Vital sign	Normal reference	Normalized	Unchanged	Became abnormal	Abnormal before procedure	Abnormal after procedure	P-value
SBP	>100	413 (15.0%)	2,091 (76.1%)	242 (8.8%)	950 (34.6%)	779 (28.4%)	<0.001
HR	60–100	544 (13.9%)	2,869 (73.6%)	487 (12.5%)	2,332 (59.8%)	2,275 (58.3%)	0.032
RR	12–20	978 (24.3%)	2,728 (67.7%)	322 (8.0%)	2,684 (66.6%)	2,028 (50.3%)	<0.001
SpO_2_	>92%	928 (30.1%)	2,007 (67.2%)	147 (4.8%)	2,070 (67.2%)	1,289 (41.8%)	<0.001

**Table 9:** Frequency and percentage of EMS assessment of procedure success. EMS: Emergency Medical Services

EMS assessment of procedure	Frequency	Percentage (%)
Successful	10,708	91.8
Not successful	379	3.2
Not recorded	582	5.0
Total	11,669	100.0

**Table 10:** Frequency and percentage of EMS assessment of patient response to the procedure. EMS: Emergency Medical Services

EMS assessment of patient response	Frequency	Percentage (%)
Improved	4,815	41.2
Unchanged	5,611	48.1
Worse	17	0.2
Not recorded	1,226	10.5
Total	11,669	100.0

**Table 11:** Recorded systolic blood pressure and oxygen saturation changes after the procedure. SBP: systolic blood pressure (mmHg); SpO_2_: oxygen saturation (%)

Abnormal vital sign	Improved	Worsened
			SBP	701 (25.5%)	456 (16.6%)
SpO_2_	1,627 (52.8%)	552 (17.9%)

**Table 12:** EMS reported changes in systolic blood pressure and oxygen saturation after the procedure. SBP: systolic blood pressure (mmHg); SpO_2_: oxygen saturation (%)

Vital sign improved on EMS global improvement	Improvement	No improvement	P-value
				SBP	523 (27.2%)	1,403 (72.8%)	0.003
SpO_2_	1,181 (56.6%)	907 (43.4%)	<0.001

## Discussion

Trauma is the leading cause of death in the United States for people under 44 years old [7]. In the setting of multisystem trauma, thoracic injuries such as pneumothoraces occur in 1-3% of major trauma and contribute to 60% of deaths [1,7-9]. Pneumothorax occurs when air enters the potential space between the lung and the chest wall within the pleural cavity. This can occur in blunt trauma through fractured ribs lacerating the visceral pleura or through a breach in the chest wall with penetrating trauma. Pressure build-up in one hemithorax pushes the mediastinal contents to the contralateral side, causing tension pneumothorax which can develop with visceral pleura disruption or tracheobronchial tree injury [1,2,5,7,10-12].

Unless adequately decompressed, an enlarging pneumothorax can rapidly lead to severe and sometimes fatal physiologic compromise affecting the respiratory and cardiac systems with reduction of preload due to compressed or twisted superior and inferior vena cavae leading to obstructive shock with circulatory arrest [10,13]. Needle decompression is a technique taught to emergency medical providers to treat patients with suspected tension pneumothorax [2]. According to ATLS guidelines, diagnosis of tension pneumothorax requires acute respiratory distress, absent unilateral breath sounds, and tracheal shift [5-7].

While the American College of Surgeons (ACS) recommends needle decompression as the first-line treatment for tension pneumothorax, there is little high-level evidence to support the practice. Many recommendations are based on case reports, case series, and expert opinions [14]. Smaller studies have failed to document increased survival with needle decompression and have expressed reservations toward the procedure based on poor results of a few patients [15-17]. Many of these smaller studies are not powered to detect survival benefits and instead demonstrate the improvement of surrogate variables such as EMS self-assessed success rate.

After needle decompression has been performed, it is difficult to determine whether the patient initially had tension pneumothorax or simple pneumothorax. If there is no simple pneumothorax, the field diagnosis was incorrect; if tension pneumothorax is still present, then field decompression failed. However, if the initial trauma workup reveals simple pneumothorax, it is difficult to determine whether this represents a treated tension pneumothorax or simple pneumothorax created by the pleural entry of the needle.

Improvement in vital signs, successful pleural cavity access, and the absence of tension pneumothorax at hospital arrival are markers of successful needle decompression [18]. Few studies report the specific improvements in vital signs that constitute successful decompression. Evidence such as a gush of air on entry, hemodynamic stability, and EMS self-reports are markers of success [19]. Our study provides objective indicators and endpoints in vital signs to predict a successful decompression based on the criteria for successful decompression, corroborating existing literature regarding using vital signs as indicators and endpoints for needle decompression [6,20].

Henry et al. showed that the success rate of needle decompression performed for hypoxia (70.5%) was notably higher than that performed for hemodynamic instability (20.3%) [21,22]. The results suggest that needle decompression is more often successful at reversing respiratory indications, e.g., hypoxia for hemodynamic indications. Our study corroborates this showing the highest improvement in SpO_2_.

Higher failure rates occur with improper placement, equipment malfunction, and insufficient knowledge of patient anatomy [1,2,7]. Many practitioners are unaware of the actual location of the ICS2-MCL and often identify this landmark as within the trauma box, a designated zone with a high likelihood of injury [12]. Another demerit that increases the chances of failure is the inaccurate subjective assessments of response by EMS personnel. In our analysis, pre-hospital providers rated the post-procedure condition as worse for only 17 (0.15%) of 8,219 patients. Our data show that the objective rate of deterioration from normal to abnormal for SBP is 8.8% and for SpO_2_ it is 4.8%. Objective deterioration was uncommon but far greater than 0.15%.

Our study showed a minor risk reduction and improvement in pulse measurement before and after the intervention. Weichenthal et al. showed no significant difference in survival between patients with prolonged versus short transport times who undergo needle thoracostomy [20]. Thus, subjective indications such as the time until hospital arrival, mechanism of injury, or EMS self-reports may not objectively improve outcomes without documented changes to vital signs. We show that the most significant of these is oxygen saturation in contact with a patient with tension physiology, which may be the best indication for needle decompression when attempted. This does not prove cause and effect, but the protective association supports the continued exercise of field needle decompression protocols.

Limitations

The retrospective nature of the study does not allow us to determine cause and effect. We were limited in our ability to evaluate long-term patient outcomes after arriving at the hospital after pre-hospital needle decompression. However, other studies have shown that pre-hospital needle decompression is associated with lower 24-hour mortality compared to emergent trauma center chest tube placement [1]. Therefore, we did not analyze variables such as patient survival, recurrent pneumothorax, or complications secondary to needle decompression.

## Conclusions

There is a discrepancy between the self-reported success of needle decompression and the changes in quantifiable vital signs. Self-assessment of success is likely inflated due to a lack of rigid criteria and insufficient follow-up. We recommend adopting specific criteria to report treatment response and recommend providing EMS personnel with better access to long-term outcomes to allow EMS agencies to build on the success shown in this analysis and improve the objectively measured benefit of their intervention.
